# The wheelchair as a full-body tool extending the peripersonal space

**DOI:** 10.3389/fpsyg.2015.00639

**Published:** 2015-05-18

**Authors:** Giulia Galli, Jean Paul Noel, Elisa Canzoneri, Olaf Blanke, Andrea Serino

**Affiliations:** ^1^Istituto di Ricovero e Cura a Carattere Scientifico, Santa Lucia FoundationRome, Italy; ^2^Laboratory of Cognitive Neuroscience, Brain Mind Institute, École Polytechnique Fédérale de LausanneLausanne, Switzerland; ^3^Center for Neuroprosthetics, School of Life Sciences, École Polytechnique Fédérale de LausanneLausanne, Switzerland; ^4^Vanderbilt Brain Institute, Vanderbilt UniversityNashville, TN, USA; ^5^Department of Neurology, University Hospital GenevaGeneva, Switzerland

**Keywords:** peripersonal space, tool use, visual spatial exploration, assistive device, embodiment

## Abstract

Dedicated multisensory mechanisms in the brain represent peripersonal space (PPS), a limited portion of space immediately surrounding the body. Previous studies have illustrated the malleability of PPS representation through hand-object interaction, showing that tool use extends the limits of the hand-centered PPS. In the present study we investigated the effects of a special tool, the wheelchair, in extending the action possibilities of the whole body. We used a behavioral measure to quantify the extension of the PPS around the body before and after Active (Experiment 1) and Passive (Experiment 2) training with a wheelchair and when participants were blindfolded (Experiment 3). Results suggest that a wheelchair-mediated passive exploration of far space extended PPS representation. This effect was specifically related to the possibility of receiving information from the environment through vision, since no extension effect was found when participants were blindfolded. Surprisingly, the active motor training did not induce any modification in PPS representation, probably because the wheelchair maneuver was demanding for non-expert users and thus they may have prioritized processing of information from close to the wheelchair rather than at far spatial locations. Our results suggest that plasticity in PPS representation after tool use seems not to strictly depend on active use of the tool itself, but is triggered by simultaneous processing of information from the body and the space where the body acts in the environment, which is more extended in the case of wheelchair use. These results contribute to our understanding of the mechanisms underlying body–environment interaction for developing and improving applications of assistive technological devices in different clinical populations.

## Introduction

Peripersonal space (PPS) is the portion of space immediately surrounding the body, where in general interactions between the individual and the environment happen. In the primate brain a specific neural network of brain areas including the posterior parietal cortex, the premotor cortex, and the putamen is dedicated to represent PPS by integrating multisensory stimuli occurring on or close to the body (Rizzolatti et al., 1997; Graziano and Cooke, 2006). Neurons in these brain areas typically respond to a tactile stimulus administered on a part of the body, and to a visual or an auditory stimulus approaching the same body part. The same brain areas are also directly implicated in motoric responses. Thus, it has been proposed that multisensory coding of PPS is important both to support defensive behavior (Graziano et al., 2002; Cooke et al., 2003; Cooke and Graziano, 2004; Stepniewska et al., 2005; Graziano and Cooke, 2006) and to guide voluntary actions directed toward objects (Rizzolatti et al., 1987; Cooke and Graziano, 2004; Fogassi and Luppino, 2005; Brozzoli et al., 2009, 2010, 2013; de Vignemont and Iannetti, 2014).

Most previous work on PPS representation has focused on hand–object interaction, studying how visual stimuli are coded in a hand-centered representation of space surrounding the hand, i.e., “peri-hand space” (Spence et al., 2004; Farne et al., 2005; Makin et al., 2007; Brozzoli et al., 2011, 2013; Gentile et al., 2011; Murray and Wallace, 2011). Research on peri-hand space contributed in highlighting the plastic properties of PPS representation, showing that the hand-centered PPS is dynamically modified as a function of the kind of interaction individuals have with their environment. In particular, converging evidence has shown that using a tool, which extends the possibilities of reaching objects in the far space, also extends the limits of hand-centered PPS (Iriki et al., 1996; Berti and Frassinetti, 2000; Farne and Ladavas, 2000; Maravita et al., 2001; Longo and Lourenco, 2006; Canzoneri et al., 2013b; Marini et al., 2014). In humans, tool use has been shown to induce plasticity after both short- and long-term learning and practice (Longo and Serino, 2012), so that perceptual and motor capacities are remapped based on the mode of tool use (Cardinali et al., 2009, 2012; Bassolino et al., 2010). Such evidence came from studies testing the effects of using a rake to reach and grasp far objects (Farne and Ladavas, 2000; Marini et al., 2014; Miller et al., 2014), a computer mouse (Bassolino et al., 2010), or the cane in blind people (Serino et al., 2007). On the basis of this evidence, it has been suggested that tools can be integrated into the representation of the upper limb so to become an extension of the arm (De Preester and Tsakiris, 2009).

Here we study the case of a special tool, the wheelchair, which does not expand the action space of the hand, but the action space of the whole body. Indeed, through manual, mechanic, or even passive manipulation, a wheelchair allows movements of the whole body in space. In the case of patients with spinal cord injury (SCI), the wheelchair is the most common tool used to overcome the limits of their interacting space due to their impairment. Wheelchair use has been shown to change SCI patient’s body image (Fuentes et al., 2013; Pazzaglia et al., 2013; Galli and Pazzaglia, 2015), in order to incorporate the wheelchair, as suggested by both influential theoretical models (Papadimitriou, 2008; Standal, 2011) and empirical studies (Arnhoff and Mehl, 1963; Higuchi et al., 2004, 2006a, 2009; Winance, 2006; Olsson, 2012; Fuentes et al., 2013; Pazzaglia et al., 2013). For these reasons, we propose that wheelchair use is an interesting model to study the relationship between actions of the whole body in space (Kannape et al., 2010; Kannape and Blanke, 2013) and plasticity in the representation of PPS around the whole body. Using the wheelchair also involves coordination and adjustment of the posture of the whole body and it affects the general sense of agency over whole body actions (Higuchi et al., 2006a).

In the present study, we assessed whether the representation of PPS changes when healthy adults use a wheelchair to move in their environment. As a proxy of full-body PPS representation we measured how tactile information on the trunk is integrated with auditory information in space. We applied a behavioral measure developed by our group to quantify the extension of the PPS around different body parts, i.e., the upper limb (Canzoneri et al., 2012, 2013a,b), the face (Teneggi et al., 2013), and more recently the trunk, which, in particular, appears to be a proxy of the extension of the full-body PPS (Grush, 2000; Blanke, 2012; Alsmith and Longo, 2014; Gentile et al., 2015). In this task, participants are requested to respond as fast as possible to a tactile stimulus administered on their trunk, while task-irrelevant sounds are presented, giving the impression of a sound source looming toward their bodies. The tactile stimulus is given at six different temporal delays from sound onset, implying that tactile information is processed when the sound is perceived at six different distances from the participant. Because we have repeatedly shown that a sound boosts tactile reaction times when presented close to, but not far from, the stimulated body part, that is within, but not outside, the PPS (Serino et al., 2007, 2011; Bassolino et al., 2010), the critical distance from the participant’s bodies where sounds affects tactile reaction time can be taken as a proxy of the boundary of PPS representation. In the present study this task was applied to measure the boundary of PPS in healthy participants before and after they performed a training consisting in using a wheelchair to navigate in the environment. Since usually the wheelchair is either actively used by the subject or operated by another person to passively move the subject, we compared the effect of active (Experiment 1) and passive (Experiment 2) use of the wheelchair on PPS extension. Finally, we also asked whether during passive exploration, visual cues related to the environment are necessary to change PPS representation, or, conversely, vestibular and motor cues related to full-body motion are sufficient; for this we compared the effects of passive wheelchair movement when participants were either blindfolded or not (Experiment 3). As visual information is usually processed from the front space, but wheelchair movements were performed both moving forward and backward, we measured PPS extension in both portion of the space, expecting a double modulation of PPS boundaries.

## Materials and Methods

### Participants

Thirty-seven right-handed healthy participants from the student population at the École Polytechnique Fédérale de Lausanne (EPFL) partook in the study (20 male, mean age 24.22, SD 4.38, range 18–34). Participants were randomly assigned to one of three Experiments, each requiring a different kind of training with the wheelchair. All participants were naïve to use a manual wheelchair prior to participating the study. One participant was removed from the analysis because of reaction times longer than 2.5 SDs in one condition, so that each experiment group consisted of 12 participants. All participants had normal tactile and auditory acuity, as well as normal or corrected-to normal sight and no psychiatric or neurological history. Participants were reimbursed for their participation in the study (20 CHF/h). All participants gave informed consent. The study was approved by the ethics committee of EPFL and was performed in accordance with the Declaration of Helsinki.

### Audio–Tactile Interaction Task

**FIGURE 1:**
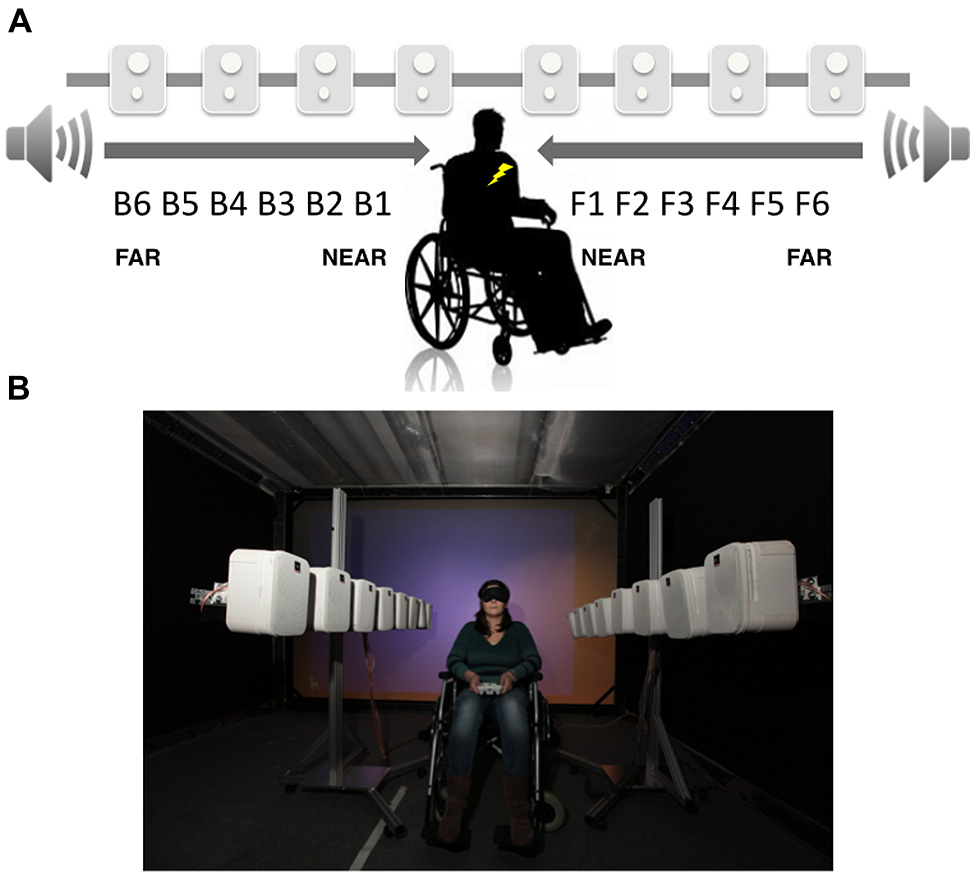
**Audio–tactile interaction task. (A)** Schematic representation of sound distances respect to the participants’ location. Participants where asked to respond as fast as possible to tactile stimulation on their trunk (symbolized by the yellow flash in figure), while two arrays of loudspeakers generated a sound stimulus starting from the far space and approaching the participants, either in the front or back space. **(B)** Picture of the experimental set up.

The procedures for the audio–tactile interaction task used to measure PPS were adapted from those described in previous studies from our group (Canzoneri et al., 2012, 2013a,b). During the task, participants were blindfolded and sat on a manual wheelchair. During audio–tactile experimental trials a sound looming toward the participant was presented. Details concerning the algorithm governing the generation of sound sources can be found on line (http://lnco.epfl.ch/labtechniques). In short, the audio rendering system producing the looming sound was composed of two longitudinal arrays of eight loudspeakers each. These two arrays were placed on the right and left side of the participants. The distance between two consecutive loudspeakers was set to 27.5 cm, and the distance between the two line arrays was set to 1 m. The loudspeakers were fixed on two independent metallic structures that allow horizontal positioning. A broadband sound source was played simultaneously through all speakers while modulating via a Gaussian function the amplitude at each specific speaker in a time dependent-manner. Participants were placed halfway between the two line arrays of speakers, as well as halfway down each array; that is, between speakers 4 and 5. This procedure allows to test PPS both in the back and front space (for a schematic representation of the set up see **Figure 1**). The stimulus generated by the loudspeakers was set to simulate a sound looming from the back until the physical location of the participant (2 m) or from the front until the physical location of the participant (2 m). A constant sound velocity of 75 cm/s was utilized. Along with the auditory stimulation, in 66% of trials, participants were also presented with a 100 ms vibrotactile stimulus placed both on the chest and on the back, at the same height and position (sternum level). Loudspeakers were placed as to match the height of the location of vibrotactile stimulation. We used two vibration devices each consisting of a small vibrating motor (Precision MicroDrives vibration motors, model 312–101, 3 V, 60 mA, 9000 rpm, 150 Hz, 5 g). The motors had a surface area (the area touching the skin) of 113 mm^2^. The devices were attached to the body using tape.

Participants were asked to respond as fast as possible to the tactile target by pressing a button with their right index finger on a response box, placed on their legs. Tactile RTs were automatically recorded. Participants were explicitly told that sounds were task irrelevant. In order to study the relationship between the position of sounds in space and their implicit effect of tactile processing, the tactile stimulus was delivered at different temporal delays from the onset of the sound, both for back and front space, so that tactile information was processed when the sound was perceived at a given distance from the participant’s body. The temporal delays for the tactile stimulus were set as follows (where B stands for Back and F for Front): for B6 and F6 tactile stimulation was administered at 380 ms after the sound onset; for B5 and F5 at 760 ms from sound onset; for B4 and F4 at 1.140 ms from sound onset; for B3 and F3 at 1.520 ms from sound onset; for B2 and F2 at 1,900 ms from sound onset; and for B1 and F1 at 2,280 ms from sound onset. In this way, the tactile stimulation occurred when the sound was perceived at different locations and 12 different sound distances were probed (B6 through B1 in the back space and F1 through F6 in the front). Set up and experimental stimuli had been already validated in a previous sound localization tasks in which sound locations were actually perceived close to the body at high temporal delays, and far from the body at low temporal delays, both for back and front space.

In the remaining trials (33% out of total), either unimodal auditory (looming sounds only) or unimodal tactile (vibration only) stimuli were administered. Unimodal auditory stimuli served as catch trials, where participants were asked to withhold response. These trials were included in order to avoid entrainment of an automatic motoric response and to assure that participants were attentive to the task. Unimodal tactile stimuli served as baseline trials. In these trials, a vibrotactile stimulus, in the absence of sounds, was delivered at the equivalent time to the nearest and furthest distance sampled during experimental trials (corresponding at temporal delays of B6 and B1 and of F1 and F6). Baseline trials were critical to demonstrate that sounds perceived within the boundaries of PPS in the experimental trials has a facilitatory effect on tactile processing, i.e., resulting in faster RT as compared to unimodal tactile trials. Baseline trials were also used to control for a potential confounding effect due to expectancy: indeed, in case of looming sounds, trial with sounds perceived closer to the body were also those trails in which the tactile target occurred later in time and thus where participants may be more prepared to respond. Overall the experiment consisted of 12 repetitions for each of the 12 spatial locations, resulting in a total of 144 critical trials with audio–tactile stimulation, randomly intermingled with 24 catch trials (auditory stimulation only) and 48 baseline trials (tactile stimulation only). Inter-trial interval was 500 ms, and each trial lasted approximately 3.66 s, for a total duration of the PPS testing of 13 min.

### Procedures

For each experiment, we measured PPS representation before and after a block of wheelchair use. The entire experiment lasted approximately 60 min and consisted of a Pre-wheelchair session of PPS assessment, a session of wheelchair use, and a Post-wheelchair session of PPS assessment.

Peripersonal space assessment before and after wheelchair use was the same for the three experiments, and consisted of the audio–tactile interaction task described above.

The wheelchair use session lasted in total approximately 13 min (i.e., the time needed by each participant to complete each action three times, which in turn was the same amount of time the audio–tactile interaction task) and was based on the wheelchair skills training program, developed by the Wheelchair Research Team (Dalhousie University and Capital Health, Halifax, NS, Canada). It consisted of a series of wheelchair actions, such as forward and backward rolls, turns while moving, turns while static in place, sideway maneuveres, passage through doors, obstacle avoidance, ascending low curbs, and parking the wheelchair (for a complete list of actions please see **Table 1**). The wheelchair use session was different for the three groups of participants. In Experiment 1, the participant maneuvered a manual wheelchair. In Experiment 2, an experimenter propelled the wheelchair the participant sat on, and finally, in Experiment 3, participants were passively propelled by the experimenter (as in Experiment 2) but were blindfolded, so to prevent visual information about the movement in the environment. During the wheelchair use session, a wide set of variables were collected and controlled for in order to exclude the possibility of wheelchair exposure confounds across experiments. In particular we evaluated the number of steps, average speed, distance and time needed to complete the obstacle course.

**Table 1:** List of the movements required for the wheelchair skills training program.

Item	Individual skill
1	Rolls forward (10 m)
2	Rolls backward (10 m)
3	Turns while moving forward (90∘)
4	Turns while moving backward (90∘)
5	Turns in place (180∘)
6	Maneuvers sideways (10 m)
7	Gets through doors and apertures
8	Rolls long distance (100 m)
9	Avoiding obstacles
10	Gets over gap and step (2 cm)

At the end of the experiment a set of questionnaires were administered to all participants in order to explore different components of their experience with the wheelchair. Some phenomenological aspects of wheelchair embodiment were assessed through an adapted version of the wheelchair embodiment questionnaire (Pazzaglia et al., 2013). Using a rating scale ranging from 0 (“completely disagree”) to 7 (“completely agree”), participants evaluated questions designed to capture the implicit and explicit aspects of tool use and embodiment. Questions included both previously adapted hypothesized constructs with prosthetic devices (Murray, 2004; Pazzaglia et al., 2013) and new *ad hoc* devised items (for a complete list of items please see **Table 2**). An adapted version of The Wheelchair Skills Test (WST, version 4.2) was also administered to verify to what extent participants felt comfortable with the wheelchair training. This test is usually adopted after rehabilitation training with patients to test their real and perceived abilities with the tool.

**Table 2:** List of the questionnaire’s items.

N	Item
1	I thought of ways to prevent problems with the wheelchair (i.e., I was paying attention to its good working/maneuvering).
2	I protected the wheelchair from dangerous maneuveres.
3	I protected myself from dangerous maneuveres.
4	I felt some kind of emotional involvement with the wheelchair.
5	I experienced some change in my attention and/or awareness while being in the wheelchair (after 5, 10, 15 min).
6	I perceived the wheelchair as an external tool.
7	I perceived the wheelchair as a part of my entire body.
8	I perceived the wheelchair as a part of my lower limbs.
9	I perceived the wheelchair as a “substitute” for my body/limbs.
10	I perceived the wheelchair as an “extension” of my body/limbs.
11	I perceived the wheelchair as a form of compensation for my actions.
12	Close your eyes and imagine yourself é (pause for 3 s). I can see the wheelchair.
13	When thinking about my body frame, I feel that the wheelchair in an internal part of my body.
14	I perceived the wheelchair as an “extension” of my reaching space.
15	I perceived the wheelchair as a “limitation” of my reaching space.
16	I perceived myself as faster.
17	I perceived myself as slower.
18	I perceived the objects around me closer.
19	I perceived the objects around me further away.
20	I perceived the objects around me easier to grasp.
21	I perceived my movements adequate and well executed.

### Data Analyses

#### Audio–Tactile Interaction Task

Since tactile stimuli were administered well above threshold, participants were extremely accurate in performing the task (all conditions 96 and 98% correct).

The performance for baseline trials and experimental multimodal trials was then analyzed in terms of reaction times (Canzoneri et al., 2012). In order to study the relationship between tactile RTs and the perceived sound position as a proxy of PPS representation, we computed mean tactile RTs at the different temporal delays for the back and front space, before and after wheelchair use. RTs for answers given before the touch was actually administered and RTs exceeding 1000 ms were discarded on single subject level. Then, RTs exceeding more than 2 SDs from the mean RT of each experiment were considered as outliers and trimmed from the analyses (1.5% of trials in total). We entered tactile RTs into a mixed model ANOVA with Condition (Pre, Post), Space (Back, Front), and Distance (D1, D2, D3, D4, D5, D6) as within-subject factors, and Experiment (Active, Passive, No-Sight) as between-subjects factor. Given the equivalent segmentation of the space at the different temporal delays (from F1 to F6, and from B6 to B1), there was a correspondence between the points (B6 corresponded to F6, B5 to F5, B4 to F4, B3 to F3, B2 to F2, and B1 to F1). Therefore, the back and the front space were considered together to study the main effect of Distance for the coupled delays. Since the critical manipulation to test PPS was to deliver the tactile stimulus when the sounds where perceived at a difference distance from the body, the critical result to show a modification of PPS representation following wheelchair use would be an interaction between Sounds and Condition. Further interactions between Sounds and Condition and Experiment would indicate whether any change in PPS was specific for the kind of wheelchair training implemented. Finally, any interaction within the previous factor and Space would indicate whether such effects were specific for the front or back space.

A significance threshold of α < 0.05 was set for all statistical analyses. All pair-wise comparisons were corrected using the Duncan’s *post hoc* test and the data are reported as the mean ± SEM.

#### Questionnaire

The questionnaire items were compared between the three groups using the Kruskal–Wallis test in order to establish whether the manipulation of wheelchair use succeeded in generating a different sense of embodiment over the tool. The data are reported as the mean ± SEM.

## Results

### Catch Trials

In order to monitor participants’ alertness and rule out the possibility that they exhibited an automatic motor response as soon as an auditory stimulus was delivered, we run a non-parametric analysis (Kruskall–Wallis *H* test) on the percentage of correct answers on the catch trials (trials in which participants heard only the auditory stimuli, but did not receive any tactile stimulation). The comparison among the three groups did not reveal significant differences in none of the conditions [Pre Back: *H* = 2.41, *p* = 0.29; χ^2^_(2)_ = 0, *p* = 1, with a mean rank score of 19 for Experiment 1, 20.5 for Experiment 2, and 16 for Experiment 3; Pre Front: *H* = 3.22, *p* = 0.20; χ^2^(2) = 0, *p* = 1, with a mean rank score of 16.4 for Experiment 1, 18.1 for Experiment 2, and 21 for Experiment 3; Post Back: *H* = 1.25, *p* = 0.53; χ^2^_(2)_ = 0, *p* = 1, with a mean rank score of 19.5 for Experiment 1, 16.5 for Experiment 2, and 19.5 for Experiment 3; Post Front: *H* = 0.37, *p* = 0.82; χ^2^_(2)_= 0, *p* = 1, with a mean rank score of 17.8 for Experiment 1, 19.5 for Experiment 2, and 18.1 for Experiment 3], meaning that all participants were equally good at withholding response when it was demanded from them, regardless to the condition or the experiment.

### Baseline Trials

Statistical analysis was conducted on the unimodal tactile trials, which served as baselines ruling out that any speeding up effect on RTs as sounds loom toward the participant could depend only on an expectancy effect. The ANOVA conducted on RTs with Condition (Pre, Post), Space (Back, Front), and Distance (D6 and D1) as within-subject factors, and Experiment (Active, Passive, No-Sight) as between-subjects factor, showed no significant main effects of Condition [*F*_(2,33)_ = 0.96, *p* = 0.33, η^2^ = 0.02]; Space [*F*_(2,33)_ = 3.74, *p* = 0.07, η^2^ = 0.10], Distance [*F*_(2,33)_ = 0.07, *p* = 0.79, η^2^ = 0.002], nor of Experiment [*F*_(2,33)_ = 0.66, *p* = 0.52, η^2^ = 0.03]. Again, none of the interactions were significant (all *p*_s_ > 0.10), meaning that any decrease in reaction times as sounds loomed toward the participant was not driven simply by an unspecific expectancy effect.

### Experimental Multimodal Trials

The critical data for measuring PPS are those from multimodal audio–tactile trials. The ANOVA conducted on RTs to multimodal trials with Condition (Pre, Post), Space (Back, Front), and Distance (D1, D2, D3, D4, D5, D6) as within-subject factors, and Experiment (Active, Passive, No-Sight) as between-subjects factor showed no significant main effect of Condition [*F*_(2,33)_ = 3.37, *p* = 0.08, η^2^ = 0.09], Space [*F*_(2,33)_ = 1.39, *p* = 0.24, η^2^ = 0.04] or Experiment [*F*_(2,33)_ = 0.88, *p* = 0.42, η^2^ = 0.05], meaning that the training did not induce any general effect on tactile RTs and all groups were equally reactive to the task. However, a significant main effect of Distance [*F*_(2,33)_ = 49.21, *p* < 0.001, η^2^ = 0.59] was found. As predicted from previous studies (e.g., Canzoneri et al., 2012), tactile RTs speeded up as soon as the sounds were perceived as being close to the body, both in the front and in the back space. Critically, the four-way (Condition × Space × Distance × Experiment) interaction approached significance [*F*_(10,165)_ = 1.78, *p* = 0.058, η^2^ = 0.10], suggesting that the different types of training induced specific effects on PPS representation. To shed light on the different modulations of PPS due to the three types of training, in the following sections, we present the analyses run for the three different experiments separately.

#### Experiment 1: Active Use of the Wheelchair

**FIGURE 2:**
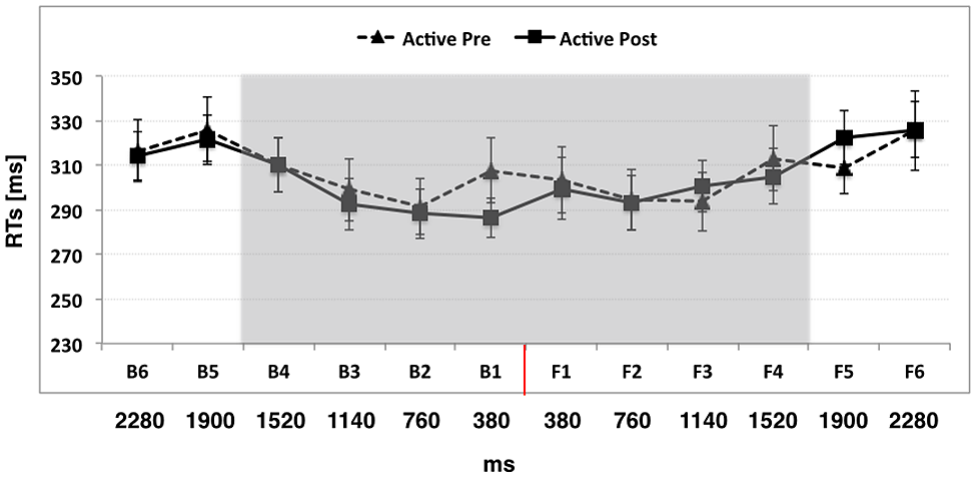
**The graph shows participants’ mean responses to the tactile target at different temporal delays from sound onset in Experiment 1 (active use of the wheelchair)**. Hatched line refers to Active Pre training condition while filled line refers to the Active Post training condition. The red line indicates the position of the subject. The shaded region indicates the boundaries of peripersonal space (PPS). Error bars denote SEM.

When participants actively used the wheelchair, the ANOVA conducted on RTs with Condition (Pre, Post), Space (Back, Front), and Distance (D1, D2, D3, D4, D5, D6) as within-subject factors showed a significant main effect of Distance [*F*_(5,55)_ = 23.99, *p* < 0.001, η^2^ = 0.68]. RTs at D6 and D5 were significantly slower than those at D4, D3, D2, and D1 (all *p*_s_ < 0.05), suggesting that the first point in space where sounds significantly boosted tactile processing was located between D4 and D5, corresponding to a distance of about 70 cm from the body, which we consider as the boundary of PPS. The two-way Distance × Space interaction was not significant [*p* = *F*_(5,55)_ = 1.28, *p* = 0.28, η^2^ = 0.10], meaning that the PPS boundary was located at the same distance in the front and in the back space. Importantly, also the two-way interaction Distance × Condition [*F*_(5,55)_ = 1.01, *p* = 0.41, η^2^ = 0.08] and the three-way interaction Distance × Condition × Space [*F*_(5,55)_ = 1.10, *p* = 0.37, η^2^ = 0.09] were not significant, meaning that active wheelchair use did not modify the boundaries of PPS. Data are reported in **Figure 2**.

#### Experiment 2: Passive Use of the Wheelchair

When participants were passively moved in space with the wheelchair, the ANOVA conducted on RTs with Condition (Pre, Post), Space (Back, Front), and Distance (D1, D2, D3, D4, D5, D6) as within-subject factors showed again a significant main effect of Distance [*F*_(5,55)_ = 14.71, *p* < 0.001, η^2^ = 0.57], compatible with a speeding effect on RT at decreasing sound distances from the body. Again, RTs at D6 and D5 were significantly slower than those at D4, D3, D2, and D1 (all *p*_s_ < 0.05), suggesting that the boundary of PPS was located at around 70 cm from the body, as in Experiment 1. We also found a main effect of Condition [*F*_(1,11)_ = 7.88, *p* = 0.01, η^2^ = 0.41], showing that participants were generally faster in the Post training session (mean RTs ± SEM, 281 ms ± 7) compared to the Pre training session (295 ms ± 9; *p* = 0.01). No main effect of Space [*F*_(1,11)_ = 0.09, *p* = 0.76, η^2^ = 0.008] was observed and none of the two-way interactions (Condition × Space; Condition × Distance; Space × Distance) were significant (all *p*_s_ > 0.10). However, and most interestingly, the three-way interaction (Condition × Space × Distance) was significant [*F*_(5,55)_ = 2.96, *p* = 0.01, η^2^ = 0.21]. In order to study this interaction, we compared RTs at each distance before and after passive wheelchair use, in the front and in the back space, separately.

In the front space, before passive use of the wheelchair the analysis revealed a significant effect of Distance [*F*_(5,55)_ = 2.54, *p* = 0.003, η^2^ = 0.18], showing that the boundary of PPS was located between F3 and F4, as RTs at F4 (306 ms ± 10) were significantly slower than RTs at F3 (284 ms ± 8; *p* = 0.01), F2 (287 ms ± 9; *p* = 0.03), and F1 (290 ms ± 6; *p* = 0.05), but not at farther distances, namely F5 (300 ms ± 9; *p* = 0.44) and F6 (301 ms ± 9; *p* = 0.49). Crucially, after passive wheelchair use, the PPS boundary shifted farther apart and was located between F4 and F5 [*F*_(5,55)_ = 11.05, *p* = < 0.001, η^2^ = 0.50], since RTs at F4 (283 ms ± 6) were now statistically faster from RTs at F5 (299 ms ± 10; *p* = 0.02) and F6 (298 ms ± 6; *p* = 0.02). This suggests that a wheelchair-mediated passive exploration of far space extended PPS representation.

In the back space, before passive use of the wheelchair the effect of distance was significant [*F*_(5,55)_ = 7.74, *p* < 0.001, η^2^ = 0.41], with the boundary of PPS located between B4 and B5, as RTs at B5 (317 ms ± 10) were significantly slower than RTs at B4 (293 ms ± 5; *p* = 0.001), B3 (288 ms ± 8; *p* < 0.001), B2 (281 ms ± 8; *p* < 0.001), and B1 (286 ms ± 6; *p* < 0.001), but not at B6 (306 ms ± 10; *p* = 0.12). Critically, after passive wheelchair use, the boundary of PPS enlarged also for the back space [*F*_(5,55)_ = 5.21, *p* = < 0.001, η^2^ = 0.32], and was located between B5 and B6, as RTs at the former distance were now faster than those at the latter distance (B5 = 287 ms ± 6 and B6 = 303 ms ± 10; *p* = 0.05).

**FIGURE 3:**
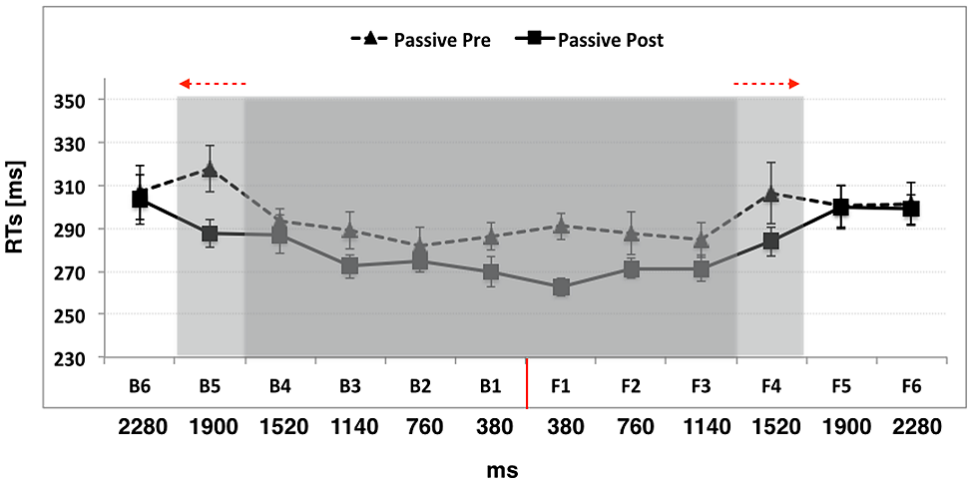
**The graph shows participants’ mean responses to the tactile target at different temporal delays from sound onset in Experiment 2 (passive use of the wheelchair)**. Hatched line refers to Passive Pre training condition while filled line refers to the Passive Post training condition. The shaded region indicates the initial boundaries of PPS (in gray) and their expansion after passive use of the wheelchair (in light gray). The red arrows indicate the direction of PPS enlargement. The red line indicates the position of the subject. Error bars denote SEM.

To sum up, before passive wheelchair use, the relationship between tactile RTs and the position of sound showed that tactile RTs sped up as the perceived sounds’ distance from the body decreased, as in Experiment 1. This spatial modulation of tactile detection due to sound position captured the boundaries of PPS representation at baseline (Canzoneri et al., 2013b), which was located approximately between B5 and B4 in the back space, i.e., corresponding to a distance of 70 cm, and between F4 and F3 in the front space, corresponding to a distance of 55 cm. After passive wheelchair use, the critical spatial range where sounds became effective in modulating tactile RTs shifted to include positions more distant from the body, that is, around B5 and F4 (i.e., at about 85 cm from the body in the back space, at about 70 cm from the body in the front space). Taken together these results are compatible with an extension of the PPS representation, both in the front and in the back space. Data are reported in **Figure 3**.

#### Experiment 3: Passive Use of the Wheelchair in Absence of Visual Information

**FIGURE 4:**
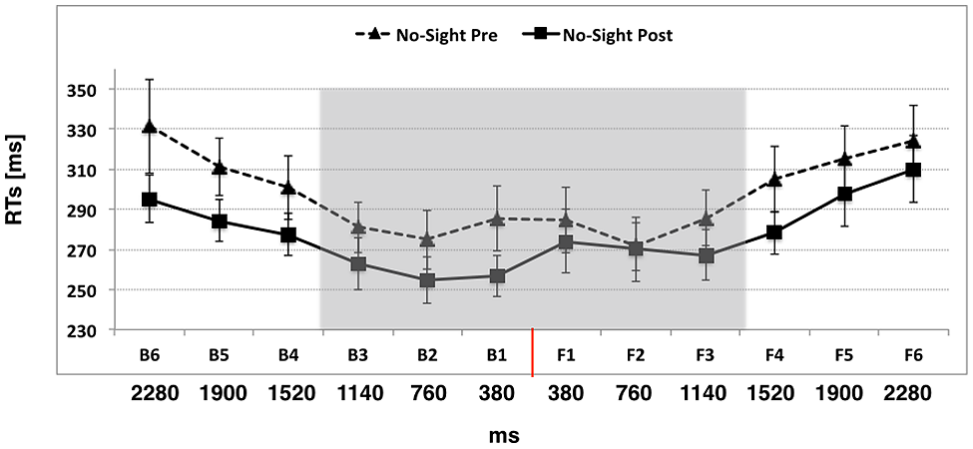
**The graph shows participants’ mean responses to the tactile target at different temporal delays from sound onset in Experiment 3 (Passive use of the wheelchair in absence of visual information)**. Hatched line refers to No-Sight Pre training condition while filled line refers to the No-Sight Post training condition. The red line indicates the position of the subject. The shaded region indicates the boundaries of PPS. Error bars denote SEM.

In Experiment 3 we asked whether visual information about the exploration of space was critical for extending PPS representation during passive use of the wheelchair, by preventing visual cues during the training. The ANOVA conducted on tactile RTs with Condition (Pre, Post), Space (Back, Front), and Distance (D1, D2, D3, D4, D5, D6) as within-subject factors, showed a significant main effect of Condition [*F*_(1,11)_ = 6.43, *p* = 0.02, η^2^ = 0.36]. Participants were generally faster in the Post training session (mean RTs ± SEM, 277 ms ± 10) compared to the Pre training session (297 ms ± 10, *p* = 0.02). The main effect of Distance was also significant [*F*_(5,55)_ = 17.11, *p* < 0.001, η^2^ = 0.60], showing that tactile RTs progressively speeded up at further proximities of the sound. RTs at D3, D2, and D1 were significantly faster than those at D4, D5, and D6 (all *p*_s_ < 0.05), implying that the boundaries of PPS was located approximately between D4 and D3, at around 50 cm from the participants body and was not affected by a passive training with the wheelchair, in absence of visual information, i.e., when no visual cues about space exploration were given to the participant (please see **Figure 4**). Importantly, differently from Experiment 2, neither the 3 way interaction Condition × Space × Distance (*p* = 0.23), nor the two-way interaction Condition × Space (*p* = 0.16) was significant. No other main effect nor interaction was significant (all *p*_s_ > 0.15).

### Questionnaires

**FIGURE 5:**
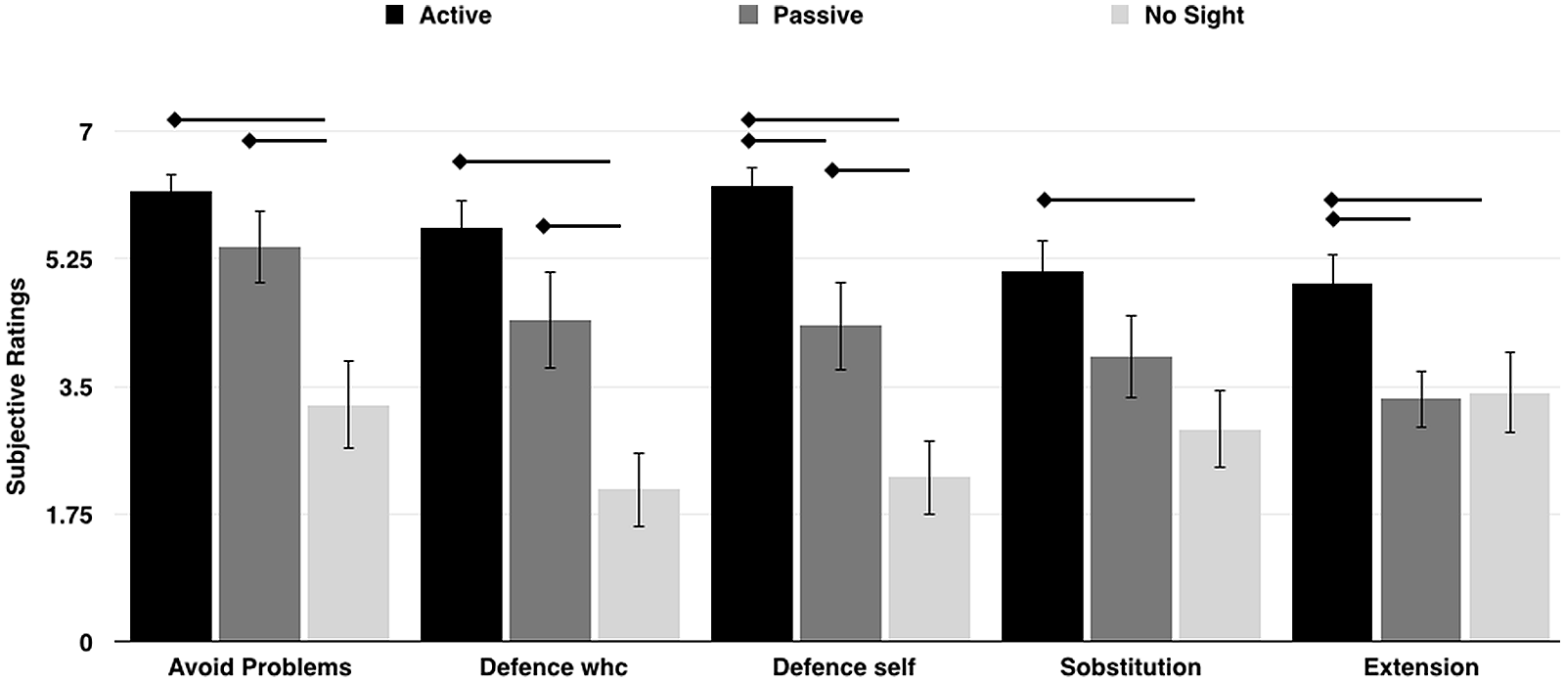
**The plot shows the mean subjective ratings for items describing functional aspects of wheelchair embodiment in the three groups of participants**. The error bars indicate the SEM. The arrows indicate significant results from the *post hoc* comparisons (*p* < 0.05).

The Kruskal–Wallis *H* test revealed significant differences across Experiments in ratings for the following items: Problem [*“I was thinking of ways to prevent problems with the wheelchair, that is I was paying attention to its good working/maneuvering”*; χ^2^(2) = 11.82, *p* = 0.003, η^2^ = 0.33, with a mean rank item score of 24.42 for Experiment 1, 20.58 for Experiment 2, and 10.50 for Experiment 3]; Defense wheelchair [*“I protected the wheelchair from dangerous maneuvering”*; χ^2^(2) = 12.98, *p* = 0.002, η^2^ = 0.37, with a mean rank item score of 25.33 for Experiment 1, 19.83 for Experiment 2, and 10.33 for Experiment 3]; Defense self [*“I protected myself from dangerous maneuvering”*; χ^2^(2) = 17.69, *p* = 0.001, η^2^ = 0.50, with a mean rank item score of 26.96 for Experiment 1, 19.29 for Experiment 2, and 9.25 for Experiment 3]; Substitution [*“I perceived the wheelchair as a substitution for my body or limbs”;* χ^2^(2) = 7.68, *p* = 0.02, η^2^ = 0.21, with a mean rank item score of 24.58 for Experiment 1, 17.96 for Experiment 2, and 12.96 for Experiment 3];, Extension [*“I perceived the wheelchair as an extension of my body or limbs”;* χ^2^(2) = 6.45, *p* = 0.04, η^2^ = 0.18, with a mean rank item score of 24.67 for Experiment 1, 14.88 for Experiment 2, and 15.96 for Experiment 3]. *Post hoc* comparisons revealed that participants in the Active and Passive group had a significantly higher rating for item Problem (*A* = 6.16 and *P* = 5.07), when compared to No-sight participants (*NS* = 3.45; *p* < 0.01 for both); as in the item Defense wheelchair (*A* = 5.66, *P* = 4.15, *NS* = 2.18; *p* < 0.01 for both) and in the item Defense self (*A* = 6.24, *P* = 4.07, *NS* = 2.36; *p* < 0.03 for both). Crucially, Active participants perceived the wheelchair more as a substitution (*A* = 5.08) and as an extension (*A* = 4.9) of their body compared to both the Passive (*P* = 3.60 and 3.15) and the No-Sight participants (*NS* = 3.09 and 3.63; all *p*_s_ < 0.05). Data are reported in **Figure 5**.

Taken together, data from questionnaires suggest that participants were more focused on their own body, rather than in exploring the space during active use of the wheelchair, which speaks in favor of a greater embodiment of the wheelchair after active use (i.e., higher scores on embodiment items of the questionnaire) compared to the passive use, both with and without visual information. This greater embodiment made active participants particularly focused on the near space. On the contrary, participants who were not directly using the wheelchair were also less prone to embody it (i.e., lower scores on embodiment items of the questionnaire), but this made them particularly receptive to the exploration of the far space.

The analyses on the other parameters and on the adapted version of the WST, (version 4.2) showed that among the three Experiments there was no difference in terms of time needed to complete the path, number of steps taken, average speed, and total distance traveled (all *p*_s_ > 0.10), ruling out any possible low level confounding variable.

## Discussion

The present study explored the role of a full-body tool, i.e., the wheelchair, in extending PPS representation and revealed three key findings, which are relevant for the understanding of the multisensory-motor basis of PPS representation and its plasticity. First, a passive exploration of space induces a modification of PPS representation, which seems to be compatible with an enlargement of PPS boundaries. Second, this effect seems to be specifically related to the possibility of receiving information from the environment through vision, as any effect due to passive exploration of the space with the wheelchair disappeared when visual information was prevented by blindfolding participants. Third, a short but intense period of motor training of active use of the wheelchair in healthy participants did not induce any modification in PPS representation.

### Passive Wheelchair Use Enhances Visual Spatial Exploration and Triggers PPS Modulation

Peripersonal space is usually conceived as a human–environment interface in which individuals plan and perform their actions (Rizzolatti et al., 1997; Brown and Goodale, 2013). To date, this space has been investigated mostly as related to the hand since it is the main body effector to interact with objects in the environment. Most of the previous studies on PPS representation and plasticity, indeed, focused on hand-object interactions coded in a hand-centered reference frames (Spence et al., 2004; Farne et al., 2005; Makin et al., 2007; Brozzoli et al., 2011, 2013; Gentile et al., 2011). However, our movements are not limited to actions of the hand or other body parts, but frequently involve movements of the whole body in space (Kannape et al., 2010; Kannape and Blanke, 2013). There are few studies that directly test whether PPS representation varies depending on full-body actions, mainly locomotion (Berti et al., 2002; Noel et al., 2014). Here we sought to examine the effect of wheelchair use that offered us the special opportunity to study at the same time a whole body pattern of action, as locomotion, in addition to the effect of tool use. This makes wheelchair use particularly interesting to be investigated, as a way to compensate for locomotion deficits and expand the action possibilities of the body as a whole. Our results suggest that even being passively propelled with the wheelchair, and thus processing information from an extended portion of space as compared to static conditions, extends, to a certain degree, PPS representation.

How is it possible that being passively propelled changes PPS representation? A commonly accepted notion is that spatial frames of reference are organizing systems supporting spatially oriented behaviors. Being passively propelled in the far space allowed participants for a greater visual exploration of the space and induced the encoding of augmented visual information synchronously coupled with the wheelchair use. We propose that the influence of vision during the training might be related, in particular, to optic flow information. Optic flow information, during locomotion, for example, has been show to induce adaptive postural changes to avoid obstacles while walking (Patla, 1998) and to affect the perceived size of an object relative to the body (Mark et al., 1990). Brain areas processing optic flow situated in the middle temporal (MT) and medial superior temporal (MST) areas (Duffy, 1998; Anderson and Siegel, 1999) directly project to posterior parietal areas (e.g., Ventral Intraparietal Area), which integrate somatosensory, visual, and auditory information in PPS and are also sensitive to optic flow (Bremmer et al., 2001, 2002). Thus, visual information related to self-motion during passive wheelchair use might be sufficient to trigger the extension effect in PPS representation. This suggestion is supported by the finding that PPS modulation was absent in participants who underwent the same spatial exploration activity, in the visually deprived condition (Experiment 3).

### Active Wheelchair Use Enhances Tool Embodiment, but Prevents Visual Spatial Exploration

If a passive exploration of the far space can trigger an expansion of the PPS representation, as shown by the results of Experiment 2, one might predict a similar, or even greater expansion in a condition of active wheelchair manipulation. Surprisingly, the active motor training with the wheelchair did not induce any modification in PPS representation.

Motor actions require the egocentric coding of space (Higuchi et al., 2006b) where the perceptual consequences of self-motion are strictly related to bodily awareness and the space is differently represented depending on the action capabilities. In our study, participants in Experiment 1 (i.e., active wheelchair use) were the agents of the wheelchair’s movements. Their ratings on the embodiment questionnaire revealed that the more participants were using the wheelchair, the more they experienced embodiment over it. At the same time, they also paid more attention related to moving the wheelchair, and how to protect it and themselves from dangerous maneuvers during active wheelchair use. These subjective reports suggest that participants in the active condition were highly body-centered and less focused on external stimuli, likely preventing the exploration of the space around them. Paying attention mostly to their body action on the wheelchair, and thus to the space immediately around the body, may have thus prevented the exploration of the far and informative space. Normally it has been found that embodied tools expand PPS representation because they allow to extending the consequences of body actions to stimuli in the far space. Here, participants from the Active group acted principally in their near bodily space. As a consequence, this may have prevented extending PPS representation toward the far space after active use of the wheelchair. Thus, greater embodiment of a tool, in this case the wheelchair, does not necessarily imply a greater extension of PPS boundaries (Bassolino et al., 2014), at least for full-body tools as a wheelchair.

This interpretation is related to another factor, which could have played a role on the null effect of active wheelchair use. Our participants were fully walking able individuals, who had not used any wheelchair before the experiment. Thus, the present training required learning of new skills to control the wheelchair. It is possible that being able to control and reliably predict a tool’s actions is necessary to change spatial representation. Tool motor learning may play a role in this modulation because it allows the user to make predictions about the spatial location of the body and tool posture at the same time. In the present case it is also possible that the active wheelchair training was not long enough to induce a modulation of PPS representation because it was not sufficient to induce enough motor expertise to trigger accurate predictions about the sensory consequences of wheelchair movements in the environment. However, this result seems to be in contrast with previous observations indicating that PPS reshapes also after short training (Serino et al., 2007; Bassolino et al., 2010; Canzoneri et al., 2013b). However, in the present case, differently from previous reports, for our participants this was the first experience with the full-body tool (wheelchair), implying levels of motor learning not necessary for hand held tools. Thus, when people are presented with standard hand-controlled tools, they are able to acquire motor control information quickly, adapting also their PPS representation (Maravita et al., 2002; Holmes et al., 2007). In contrast, learning how to operate a whole-body wheelchair, which implies unusual spatial mappings and which is also unfamiliar in terms of spatial and temporal dynamics, requires participants to put considerable amounts of effort and attentional resources in the adaptation to the new motor behavior and extensive practice. This effect might have prevented them from visual spatial exploration required for modulating PPS representation. Even if adaptation can occur quickly and accurately in response to other artificially altered conditions, it is likely that the adjustment would take a much longer time when the form of locomotion changes dramatically from walking to wheelchair (Higuchi et al., 2004) or orthoses (van Hedel and Dietz, 2004) use.

### Reconciling Null and Positive Effects of Active vs. Passive Wheelchair Use

The absence of a modulation of PPS representation in the active condition, and the presence of such effect in the passive condition can be interpreted in the light of a computational neural network model developed to explain neural mechanisms of PPS representation and plasticity (Magosso et al., 2010a,b). According to this model, PPS extension after tool-use does not depend on the tool itself, but it is a consequence of pairing tactile stimulation at the hand location (by handling the tool) with synchronized visual or auditory stimuli occurring in the far space (where the tool exerts its effects). Thus, the model predicts that simply presenting tactile near stimuli and synchronous visual (or auditory) far stimuli, independently from any tool use, would be sufficient to extend PPS representation. On the contrary, no PPS extension is predicted in case of asynchronous tactile and visual or auditory stimulation. This prediction was confirmed both by simulation and behavioral experiments reported recently by our group (Serino et al., 2015). In the present study, participants in the Active group processes a great amount of stimuli on their physical body, which were not systematically coupled with external/far visual stimuli, because participants had to focus on their own body and actions, as a consequence of the motor training. On the contrary in the Passive condition, the coupling between stimuli on the body (for instance, leaning in the wheelchair) with the stream of visual information coming from the external world was stronger and synchronized. Being less focused on moving the wheelchair, passive group participants may have dedicated more attentional resources to external stimuli, looked around during the training and explored the environment, while still processing somatosensory and vestibular cues from their body. We argue that such synchronous stimulation of the body and from the far space was sufficient to extend the PPS representation. Indeed, if vision of the environment was prevented, as in Experiment 3, no extension of PPS occurred.

## Conclusion

To conclude, findings from the present study suggest that the wheelchair can be conceived as a whole body tool, enabling extended interaction between the person and the environment, thus extending PPS boundaries. However, counter-intuitively, such effect was not induced by active use of the wheelchair in healthy participants who never used a wheelchair before (even if they subjectively reported to “embody” the wheelchair). We argue that this was the case because the participants were focused on the motor training and in processing information immediately related to the wheelchair and their body and thus on stimuli coming from near space. In contrast, participants who were passively propelled on the wheelchair were able to integrate information from the external environment and their body that triggered PPS extension. Taken together, these findings offer empirical support to the hypothesis that plasticity in PPS representation after tool use does not strictly depend on the active use of the tool itself, but it is triggered by the coupled multisensory stimulation related of the physical body and to the external space, which in turn depends on the motor activity and motor expertise of the person. It is possible, for instance, that in expert wheelchair users, active use of the wheelchair does not require high attentional resources for motor control and for stimuli in the near space, allowing them to process stimuli from the far space, in a proactive way. As a consequence, PPS representation should extend in expert wheelchair users when they actively navigate with their wheelchair, differently from non-expert users in the present study. Understanding these mechanisms might be important for developing and improving applications of assistive technological devices in different clinical populations.

## Conflict of Interest Statement

The authors declare that the research was conducted in the absence of any commercial or financial relationships that could be construed as a potential conflict of interest.
